# RHO-Associated Coiled-Coil-Containing Protein Kinase Inhibitors Significantly Modulate the Epithelial–Mesenchymal Transition Induced by TGF-β2 in the 2-D and 3-D Cultures of Human Corneal Stroma Fibroblasts

**DOI:** 10.3390/biomedicines12122784

**Published:** 2024-12-06

**Authors:** Araya Umetsu, Yosuke Ida, Tatsuya Sato, Megumi Higashide, Nami Nishikiori, Masato Furuhashi, Hiroshi Ohguro, Megumi Watanabe

**Affiliations:** 1 Department of Ophthalmology, School of Medicine, Sapporo Medical University, S1 W16 Chuo-ku, Sapporo City 060-8543, Hokkaido, Japan; araya.umetsu@sapmed.ac.jp (A.U.);; 2 Department of Cardiovascular, Renal and Metabolic Medicine, School of Medicine, Sapporo Medical University, S1 W16 Chuo-ku, Sapporo City 060-8543, Hokkaido, Japan; 3 Department of Cellular Physiology and Signal Transduction, School of Medicine, Sapporo Medical University, S1 W16 Chuo-ku, Sapporo City 060-8543, Hokkaido, Japan

**Keywords:** three-dimensional spheroid culture, TGF-β2, human corneal stroma fibroblasts (HCSFs), corneal injury model, ROCK inhibitor

## Abstract

Background/Objectives: The objective of the present study was to examine the unidentified effects that RHO-associated coiled-coil-containing protein kinase 1 and 2 antagonists exert on the transforming growth factor beta2-induced epithelial–mesenchymal transition of the human corneal stroma. Methods: In the presence or absence of pan-RHO-associated coiled-coil-containing protein kinase inhibitors, ripasudil or Y27632 and RHO-associated coiled-coil-containing protein kinase 2 inhibitor, KD025, we analyzed the following: (1) planar proliferation caused by trans-endothelial electrical resistance and the cellular metabolic characteristics of the two-dimensional cultures of human corneal stroma fibroblasts; (2) the physical properties of a three-dimensional human corneal stroma fibroblasts spheroid; and (3) the gene expressions and their regulators in the extracellular matrix, along with the tissue inhibitors of metalloproteinases and matrix metalloproteinases and the endoplasmic reticulum stress-related factors of the two-dimensional and three-dimensional cultures in human corneal stroma fibroblasts. Results: Exposure to 5 nM of the transforming growth factor beta2 markedly increased the trans-endothelial electrical resistance values as well as the metabolic function in two-dimensional cultures of human corneal stroma fibroblasts. With an increase in stiffening, this exposure also reduced the size of three-dimensional human corneal stroma fibroblast spheroids, which are typical cellular phenotypes of the epithelial–mesenchymal transition. Both pan-RHO-associated coiled-coil-containing protein kinase inhibitors and RHO-associated coiled-coil-containing protein kinase 2 inhibitors substantially modulated these transforming growth factor beta2-induced effects, albeit in a different manner. Gene expression analysis supported such biological alterations via either with transforming growth factor beta2 alone or with the RHO-associated coiled-coil-containing protein kinase inhibitors variants with the noted exception being the transforming growth factor beta2-induced effects toward the three-dimensional human corneal stroma fibroblast spheroid. Conclusions: The findings presented herein suggest the following: (1) the epithelial–mesenchymal transition could be spontaneously evoked in the three-dimensional human corneal stroma fibroblast spheroid, and, therefore, the epithelial–mesenchymal transition induced by transforming growth factor beta2 could differ between two-dimensional and three-dimensional cultured HCSF cells; and (2) the inhibition of ROCK1 and 2 significantly modulates the transforming growth factor beta2-induced an epithelial–mesenchymal transition in both two-dimensionally and three-dimensionally cultured human corneal stroma fibroblasts, albeit in a different manner.

## 1. Introduction

Among six different anatomical layers, the epithelium, the Bowman’s membrane, the stroma, Dua’s layer, the Descemet membrane, and the endothelium, the stroma layer occupied approximately 90% of the thickness of the cornea [1,2]. The primary role of keratocyte is to secrete extracellular matrix (ECM) proteins such as collagens, lumican, and keratocan [3], and, thus, the regulatory mechanism affecting expressions of the ECM proteins is extremely important to maintain the transparent cornea under normal physiological conditions. Transforming growth factor-β (TGF-β) signaling is one of the pivotal pathways responsible for inducing the epithelial–mesenchymal transition (EMT) known as critical mechanisms [4,5,6,7,8] to induce the production of disorganized ECMs in corneal stromal layers, resulting in corneal stromal opacity and fibrosis. Therefore, it has been suggested that the EMT in corneal stroma is, in turn, recognized as a possible therapeutic strategy to prevent corneal opacities. However, suitable in vitro models had not been developed to study these issues. To establish reliable in vitro models replicating the EMT in the corneal stroma, the two- and three-dimensional (2-D and 3-D) cultures of human corneal stroma fibroblasts (HCSFs) were subjected to various morphological and physiological measurements in the presence of TGF-β2 [9]. In the 2-D culture of HCSFs, a significant increase in ECM deposits, an increase in planar proliferation and increases in both mitochondrial and glycolytic functions were observed in a TGF-β2 concentration-dependent manner. In the 3-D HCSF spheroids however, TGF-β2 caused downsizing and stiffening, but the TGF-β2-induced modulations of the gene expressions of evaluated molecules were significantly less compared with those of 2-D HCSF cells. Therefore, based on these collective findings that TGF-β2-induced effects on 2-D-cultured HCSF cells were significantly different from that of 3-D HCSF spheroids, it was suggested that 3-D HCSF spheroids could replicate EMT changes in a spatial environment of corneal stroma, and this may become a suitable in vitro model of the EMT in corneal stroma.

Actin filament and stress fiber assembly are known to be associated during a transition of keratocytes to fibroblasts [10,11] and are regulated by an RHO-associated coiled-coil-containing protein kinase (ROCK) signaling [12,13], which induces a variety of physiological roles, including chemotaxis, neural cell growth, and muscle contraction [14,15,16] by assembly and the organization of actomyosin filaments [17,18,19] as well as pathological roles in several ocular diseases, including corneal dysfunction, glaucoma, cataracts, and retinopathy [20,21,22,23,24]. Based on this evidence, it has been suggested that ROCKs are possible therapeutic targets for these ocular diseases, and, in fact, a pan-ROCK inhibitor (pan-ROCK-i) ripasudil (Rip) has been used as a hypotensive agent for patients with glaucoma and ocular hypertension [25]. In addition, it has been shown that ROCK-i also induces wound healing in the corneal epithelium [13,26] as well as in the corneal stroma [27]. Furthermore, in our recent study using 2- and 3-D HCSFs, Rip and ROCK2 inhibitor (ROCK2-i), KD025, it was suggested that ROCK1 and 2 are differentially involved in the spatial construction of 3-D HCSF spheroids [28]. Collectively, the TGF-β2-stimulated effects in our developed in vitro models of human corneal stroma (HCS) using the 2- and 3-D cultures of HCSFs have generated great interest in the study of the effects induced by ROCK-is toward those models.

In the present study, to characterize the ROCK-is evoked effects toward the TGF-β2-stimulated corneal stromal EMT, using in vitro two-dimensional (2-D) and 3-D spheroid culture models using HCSFs [28], various analyses by (1) trans-endothelial electrical resistance (TEER) (2-D); (2) real-time cellular metabolic measurement (2-D); (3) 3-D spheroids’ size and stiffness measurements; and (4) qPCR analysis for ECM proteins, their modulators, and endoplasmic reticulum (ER) stress-related factors (2- and 3-D).

## 2. Materials and Methods

The current study was carried out complying with the tenets of the Declaration of Helsinki and national laws for the protection of personal data at Sapporo Medical University Hospital after receiving approval from the institutional review board (IRB registration number 282-8). Informed consent from all subjects who participated in this study was obtained.

【Two-dimensional (2-D) and three-dimensional (3-D) cultures of human corneal stroma fibroblasts (HCSFs)】

HCSFs were surgically collected from dissected HCS specimens obtained from 2 patients with traumatic corneal injuries as described previously [29]. In brief, corneal epithelial and endothelial layers were gently removed, and the remaining corneal stromal segments, which were dissected into approximately 2–5 mm small pieces, were placed on 150 mm culture dishes and submerged in a growth medium (high-glucose Dulbecco’s Modified Eagle Medium (HG-DMEM) containing 10% fetal bovine serum (FBS), 1% L-glutamine, and 1% antibiotic-antimycotic). Those corneal stromal explants were cultured in a humidified cell culture incubator (at 37 °C with 5% CO_2_) with a change in culture medium every 2 to 3 days. The HCSF cells that sprouted around corneal stromal explants were collected and further cultured and maintained until they reached 90% confluence at 37 °C in the growth medium via daily medium exchange. The obtained HCSF was characterized by a phase contrast microscopy (ECLIPSE Ts 2, Nikon, Tokyo, Japan) and cell viability by using a commercially available kit (Cell Counting Kit-8, Dojindo, Tokyo, Japan).

The 3-D spheroid culture of the HCSFs was carried out using a hanging droplet spheroid three-dimension culture system as reported previously [30]. Briefly, after washing 2-D cultured HCSFs with phosphate-buffered saline (PBS), the cells were collected by using 0.25% Trypsin/EDTA and following centrifugation for 5 min at 300× *g*. The cell pellet was re-suspended in a 3-D culture medium composed of 2-D growth medium supplemented with 0.25% methylcellulose (Methocel A4M). Cell numbers of HCSFs were adjusted approximately as 20,000 in 28 μL of a 3-D spheroid medium and placed to each well of the culture plate (# HDP1385, Sigma-Aldrich, Burlington, MA, USA). Subsequently, the 3-D spheroid culture was maintained by the daily medium changing of half-volume until Day 6. For the evaluation of effects of ROCK-is: ripasudil (Rip) (generous gift from Kowa Company Ltd., Nagoya, Japan), Y27632 and KD025; 10 μM concentrations of them were administered.

【Trans-endothelial electron resistance (TEER) measurement of the 2-D-cultured HCSF monolayers】

The TEER measurements of the 2-D-cultured HCSF cell monolayers were conducted according to previously described methods [31]. Briefly, in a typical run, the 2-D-cultured HCSF cells were seeded onto 12-well plates for TEER plates (12 wells, 0.4 μm pore size and 12 mm diameter; Corning Transwell, Sigma-Aldrich) at a density of 2.0 × 10^4^ cells per well. In each well, inside of the membrane inserts and outside of the membrane inserts were maintained in 0.5 and 1.5 mL of the growth culture medium, respectively. When the cells reached approximately 80% confluence, 5 nM of TGF-β2 and/or 10 μM of ROCK-i (Rip, Y27632 or KD025) was added to the inside of the membrane inserts (Day 1). The culture medium of the inside of the membrane inserts was changed every other day. TEER values (Ωcm^2^) were measured by using an electrical resistance system (KANTO CHEMICAL CO. INC., Tokyo, Japan) following to the manufacturer’s instructions after washing twice with PBS.

【Measurement of real-time cellular metabolic functions】

Measurements of the oxygen consumption rate (OCR) and extracellular acidification rate (ECAR) of 2-D cultured 2-D HCSFs that were treated with or not treated with TGF-β2 and/or ROCK-is were carried out using a Seahorse XFe96 Bioanalyzer (Agilent Technologies, Santa Clara, CA, USA) as described in our previous report [32]. Briefly, approximately 2.0 × 10^4^ of the 2-D HCSFs were subjected in the wells of a 96-well assay plate (#103794-100, Agilent Technologies, Santa Clara, CA, USA) and incubated at 37 °C for 24 h. Then, the culture medium was exchanged with the XF DMEM assay medium (pH 7.4, #103575-100, Agilent Technologies, Santa Clara, CA, USA) supplemented with glucose (5.5 mM), sodium pyruvate (1.0 mM), and glutamine (2.0 mM), and the plate was incubated for 1 h in a CO_2_-free incubator at 37 °C. Measurements of the OCR and ECAR were carried out using an XFe96 Bioanalyzer with the sequential injection of oligomycin (2.0 μM), carbonyl cyanide p-trifluoromethoxyphenylhydrazone (FCCP, 5.0 μM), a mixture of rotenone (1.0 μM) and antimycin A (1.0 μM), and 2-deoxyglucose (2DG, 10 mM). Normalization of the values of the OCR and ECAR was performed using the amounts of protein assessed using a BCA protein assay (TaKaRa, Tokyo, Japan) per well by lysing the cells of the wells using a Cell Lytic buffer (Sigma-Aldrich, #C3228, Darmstadt, Germany): Basal Respiration: subtraction of the OCR with rotenone/antimycin A from the OCR at baseline; ATP-linked Respiration: the difference in the OCR after the addition of oligomycin; Proton Leak: subtraction of the OCR with rotenone/antimycin A from the OCR after the addition of oligomycin; Maximal Respiration: subtraction of the OCR with rotenone/antimycin A from the OCR after the addition of FCCP; Spare Respiratory Capacity: subtraction of the OCR at baseline from the OCR after the addition of FCCP; Non-mitochondrial Respiration: the OCR with rotenone/antimycin A; Basal ECAR: subtraction of the end point of ECAR after the injection of 2-DG from the ECAR at the baseline; Glycolytic Capacity: subtraction of the end point of the ECAR after the injection of 2-DG from the ECAR with oligomycin; Glycolytic Reserve: subtraction of the ECAR at baseline from the ECAR with oligomycin; Non-glycolytic Acidification: the end point of the ECAR after the injection of 2-DG.

【Quantitative PCR】

The extraction of total RNA following reverse transcription using an RNeasy mini kit (Qiagen, Valencia, CA, USA) and a quantitative real-time PCR (qRT-PCR) using the SuperScript IV kit (Invitrogen, Waltham, MA, USA) were carried out as previously reported using specific primers and probes (Supplementary Table S1). The normalization of each respective gene expression was compared with the expression of internal control 36B4 (Rplp0).

【Measurement of the physical properties, size, and stiffness of 3-D HCSF spheroids】

Configurations of the 3-D spheroids were observed by using a phase contrast (PC, Nikon ECLIPSE TS2; Tokyo, Japan). For the measurement of the mean size of each 3-D spheroid, the largest cross-sectional area (CSA) of each 3-D spheroid was determined using Image-J software version 1.51n (National Institutes of Health, Bethesda, MD, USA) [30].

For the evaluation of stiffness of the 3-D spheroids, a single living spheroid was directly compressed until achieving a spheroid with 50% of their diameter during 20 s by using a micro-squeezer (MicroSquisher, CellScale, Waterloo, ON, Canada) equipped with a monitor camera, as recently reported [30]. The requiring force (μN) was measured, and force/displacement (μN/μm) was used as a spheroid stiffness index.

【Statistical analysis】

Using Graph Pad Prism 9 (GraphPad Software, San Diego, CA, USA), all statistical analyses were carried out. To compare two mean values, a two-tailed Student’s *t*-test was used. For analysis of the difference in groups, a two-way analysis of variance (ANOVA) was used, followed by Tukey’s multiple comparison test. Data are shown as the mean ± standard error of the mean (SEM).

## 3. Results

We studied the ROCK inhibitory effects toward the TGF-β2-induced EMT of the corneal stroma treated and not treated with 10 μM of ROCK-i (pan-ROCK inhibitor Rip and Y27632, as well as ROCK2 selective inhibitor, KD025). The objective is to establish the EMT-related aspects, planar proliferation, cellular metabolic functions of 2-D-cultured HCSF cells, physical properties of the 3-D HCSF spheroids, and several possible molecules related to cell construction such as ECM proteins, their regulators, and ER stress-related factors of both 2-D and 3-D cultured HCSFs.

**Figure 1:**
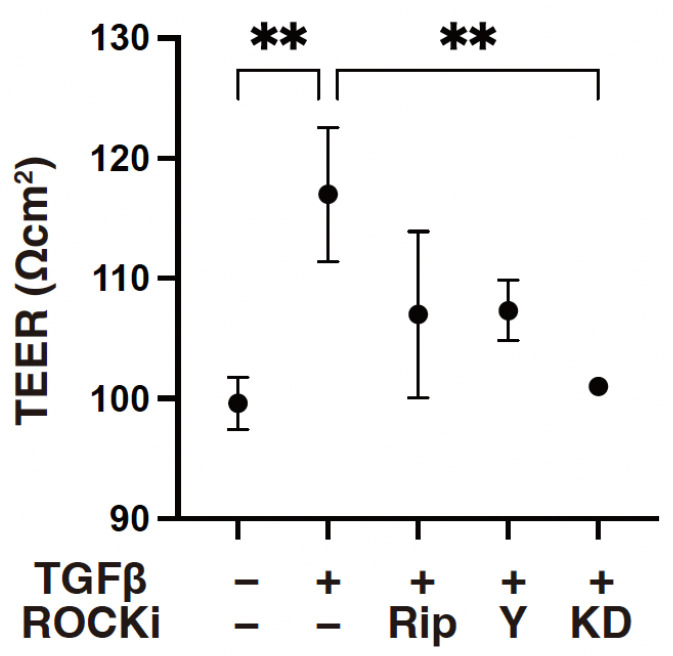
Effects of ROCK-is on the planar proliferation of human corneal stroma fibroblasts (HCSFs) evaluated by transendothelial electrical resistance (TEER). In both the administered or not administered with 10 μM of ROCK-i (Rip, Y27632 or KD025), 5 nM of TGF-β2-treated 2-D HCSF monolayers were generated during a 6-day culture. The TEER values (panel A) were plotted. Experiments were carried out in duplicate using fresh preparations (*n* = 5, each). ** *p* < 0.01.

As shown in Figure 1, the 5 nM TGF-β2-induced substantial increases in the planar proliferation were estimated via TEER, and those were either relatively or significantly inhibited by the 10 μM of pan-ROCK-i (Rip or Y27632) or ROCK2-i (KD025), respectively.

**Figure 2:**
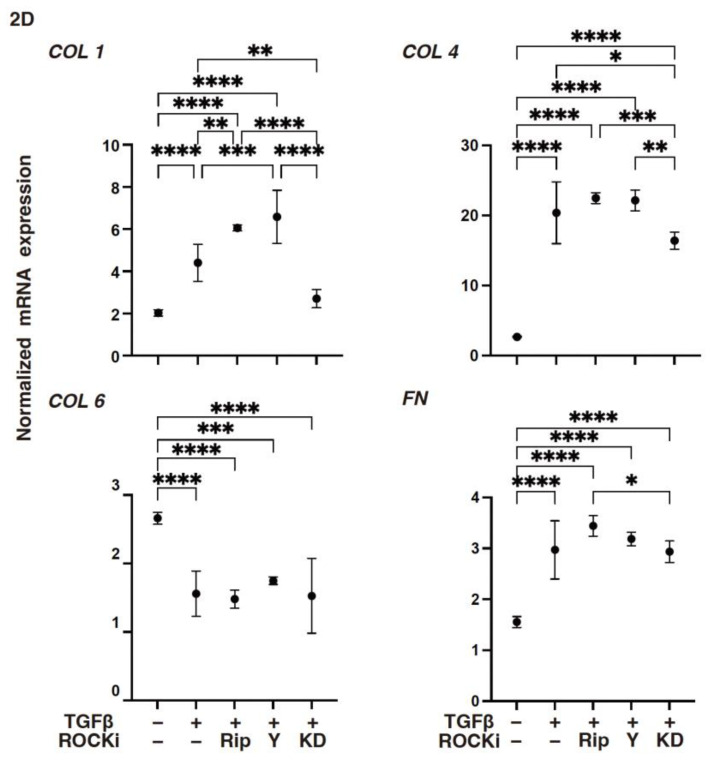
The mRNA expression in ECM proteins in TGF-β2-treated 2-D-cultured human corneal stroma fibroblasts (HCSFs) in the absence or presence of ROCK-is. In both the administered or not administered with 10 μM of ROCK-i (Rip, Y27632 or KD025), 5 nM of TGF-β2-treated 2-D cultured HCSF monolayers were generated during a 6-day culture and subjected to a qPCR analysis for *COL1*, *COL4*, *COL6*, and *FN*. Levels of the mRNA expression were plotted. Experiments were carried out in duplicate using fresh preparations (*n* = 5, each). * *p* < 0.05, ** *p* < 0.01, *** *p* < 0.005, **** *p* < 0.001.

**Figure 3:**
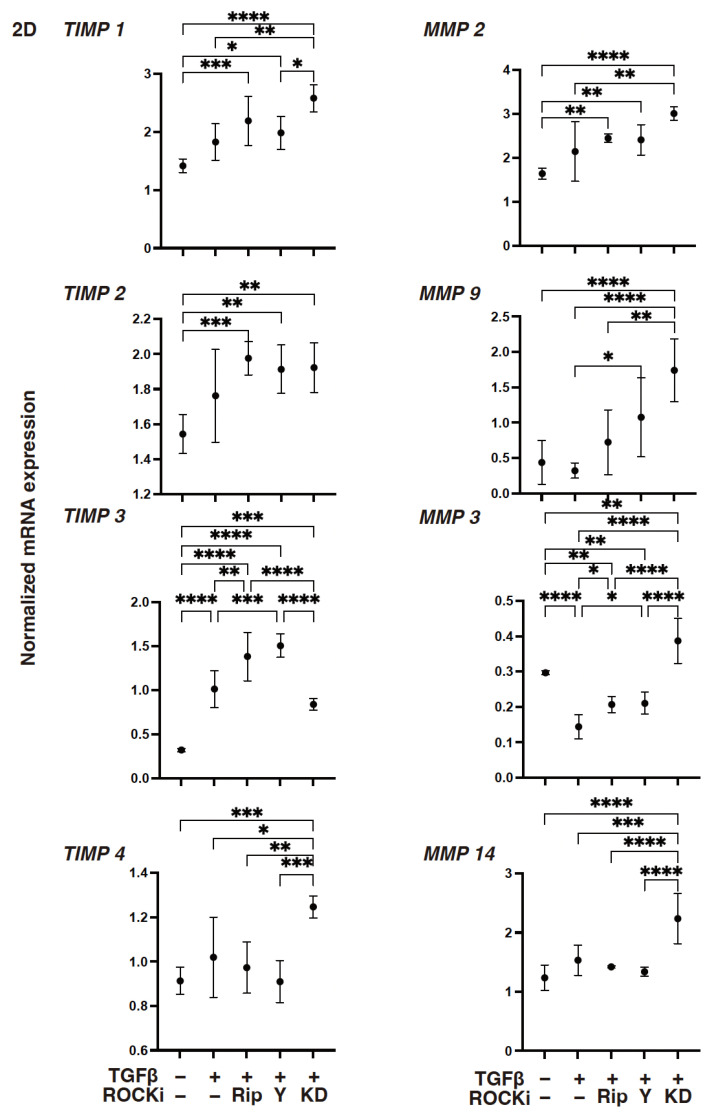
The mRNA expression of *TIMPs (1–4)* and *MMPs (2*, *3*, *9*, *14)* in TGF-β2-treated 2-D-cultured human corneal stroma fibroblasts (HCSFs) in the absence or presence of ROCK-is. In both the administered or not administered with 10 μM of ROCK-i (Rip, Y27632 or KD025), 5 nM of TGF-β2-treated 2-D-cultured HCSF monolayers were prepared during a 6-day culture and subjected to a qPCR analysis for *TIMPs (1*–*4)* and *MMPs (2*, *3*, *9*, *14)*. Levels of the mRNA expression were plotted. Experiments were carried out in duplicate using fresh preparations (*n* = 5, each). * *p* < 0.05, ** *p* < 0.01, *** *p* < 0.005, **** *p* < 0.001.

**Figure 4:**
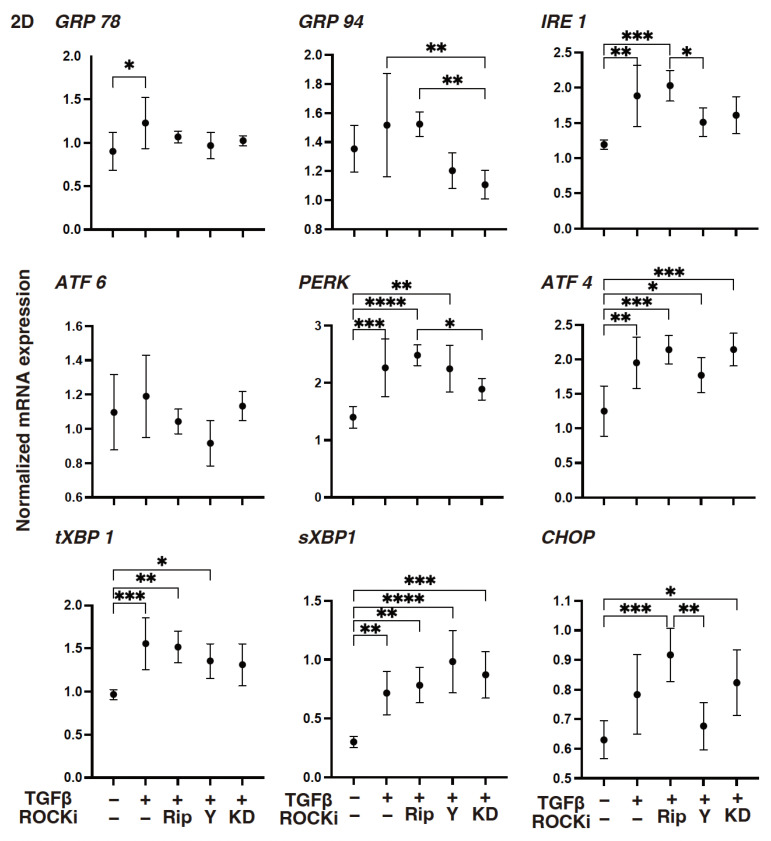
The mRNA expression of several ER stress-related genes in TGF-β2-treated 2-D-cultured human corneal stroma fibroblasts (HCSFs) in the absence or presence of ROCK-is. In both the administered or not administered with 10 μM of ROCK-i (Rip, Y27632 or KD025), 5 nM of TGF-β2-treated 2-D-cultured HCSF monolayers were prepared during a 6-day culture and subjected to qPCR analysis to estimate the mRNA expressions of several ER stress-related genes. Levels of the mRNA expression were plotted: PERK: protein kinase RNA-like endoplasmic reticulum kinase; ATF6: activating transcription factor 6; IRE1: the inositol-requiring enzyme 1; GRP78: the glucose regulator proteins 78; GRP94: the glucose regulator proteins 94; XBP1: the X-box binding protein-1; sXBP1: a spliced XBP1; and CHOP: the CCAAT/enhancer-binding homologous protein. Experiments were carried out in duplicate using fresh preparations (*n* = 5, each condition). * *p* < 0.05, ** *p* < 0.01, *** *p* < 0.005, **** *p* < 0.001.

To support these TGF-β2- and ROCK-i-induced effects, the levels of gene expressions of (1) *COL1*, *COL4*, and both the *FN* and *COL6* were either substantially increased or decreased, respectively (Figure 2): (2) *TIMP3* or *MMP3* were either increased or decreased, respectively (Figure 3); (3) most of the ER stress-related factors, with the exceptions of *GRP94*, *ATF6*, and *CHOP*, were significantly increased (Figure 4) upon exposure to TGF-β2. These TGF-β2-induced changes were also greatly modulated by ROCK2-i, KD025, rather than by pan-ROCK-i; that is, KD025 either induced a significant decrease or increase in the levels of mRNA expressions in *COL1*, *COL4*, and *GRP94* as well as *TIMP1* and *4*, and by *MMP2*, *3*, *9* and *14*, respectively, although both Rip and Y27632 induced a significant increase in the expression levels of *COL1*, *TIMP3*, and *MMP3*, as well as *MMP2*, *3*, and *9*, respectively (Figure 2, Figure 3 and Figure 4).

**Figure 5:**
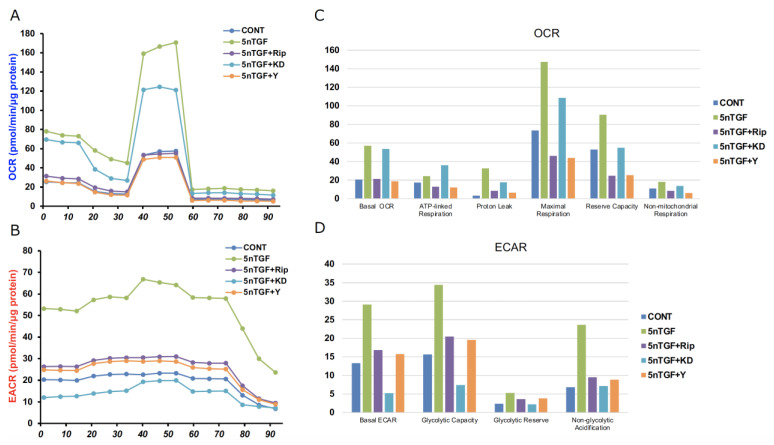
The real-time cellular metabolic functions in TGF-β2-treated 2-D planar-cultured human corneal stroma fibroblasts (HCSFs) in the absence or presence of ROCK-is. In both the administered or not administered with 10 μM of ROCK-i (Rip, Y27632 or KD025), 5 nM of TGF-β2-treated 2-D-cultured HCSF monolayers were prepared during a 6-day culture and subjected to a Seahorse real-time metabolic function analysis. Panel (**A**) shows OCR: the rate of oxygen consumption, and Panel (**B**) shows ECAR: the extracellular acidification rate. The key parameters of mitochondrial respiration and glycolytic flux are shown in panels (**C**,**D**), respectively. All experiments were performed using fresh preparations (*n* = 6). *p* < 0.05.

Both mitochondrial and glycolytic functions were also significantly enhanced by TGF-β2 as reported in our recent report [9], and those effects were markedly modulated by ROCK-i (Figure 5).

However, it is interesting that such ROCK-i-induced effects toward glycolytic or mitochondrial functions were different. In other words, either the former or the latter was predominantly suppressed by pan-ROCK-i, Rip or Y27632, as well as by ROCK2-i, KD025, respectively. Collectively, these results indicate that the suppressive effects toward TGF-β2-induced planar proliferation and glycolytic functions or mitochondrial functions in the 2-D-cultured HCSF cells were predominantly responsible for both the ROCK2- and ROCK1-related mechanisms, respectively.

**Figure 6:**
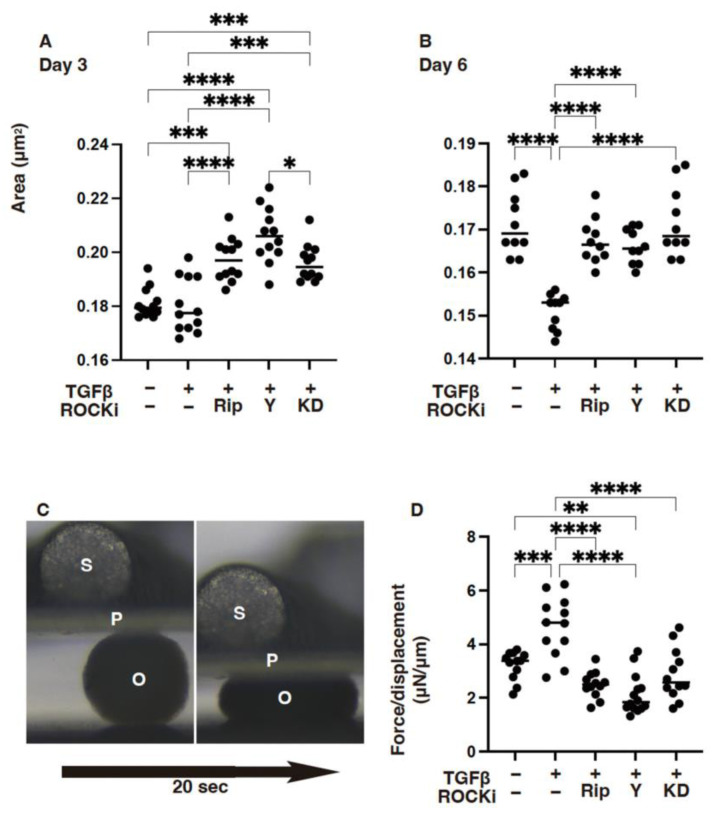
The mean sizes of the 3-D HCSF spheroids on Day 3 (**A**), on Day 6 (**B**), and the physical solidity of the TGF-β2-treated 3-D HCSF spheroids on Day 6 (**C**,**D**) in the absence or presence of ROCK-is. In both the administered or not administered with 10 μM of ROCK-i (Rip, Y27632 or KD025), 5 nM of TGF-β2-treated 3-D HCSF spheroids were prepared during a 6-day culture and subjected to physical property measurements: Panel (**A**) plots of mean sizes at Day 3; Panel (**B**) plots of mean sizes at Day 6; Panel (**C**) stiffness analysis by a micro-squeezer (S: sensor, P: pressing plate. O: spheroid); and Panel (**D**) plots of force/displacement (μN/μm) values. Experiments were carried out in duplicate using fresh preparations (*n* = 15, each). * *p* < 0.05, ** *p* < 0.01, *** *p* < 0.005, **** *p* < 0.001.

Next, to elucidate further the effects of ROCK-is (Rip, Y27632 and KD025) toward the TGF-β2-affected 3-D corneal stromal architecture as additional aspects of the EMT and the physical aspects, sizes, and stiffness of the 3-D spheroid, the HCSF was studied. As shown in Figure 6, we found that 5 nM of TGF-β2 induced significant downsizing and harder 3-D HCSF spheroids, but TGF-β2 induced effects that were diminished by either of these forms of ROCK-is.

**Figure 7:**
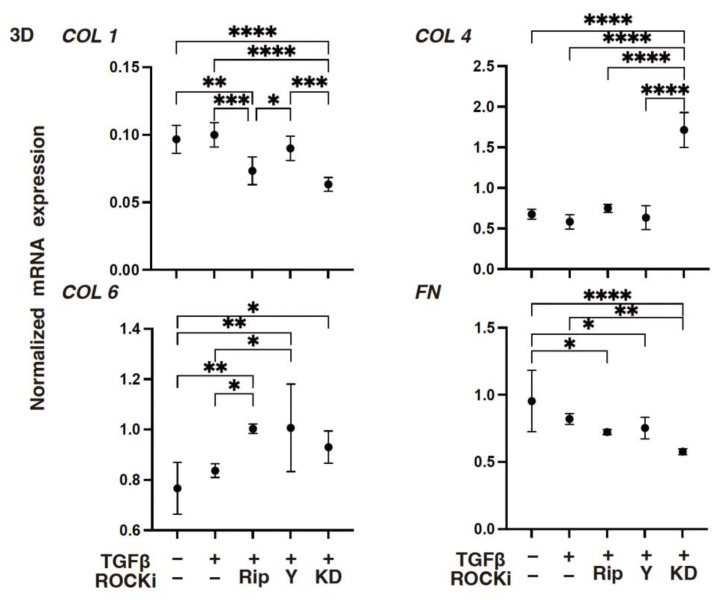
The mRNA expression of major ECMs in TGF-β2-treated 3-D-cultured human corneal stroma fibroblasts (HCSFs) in the absence or presence of ROCK-is. In both the administered or not administered with 10 μM of ROCK-i (Rip, Y27632 or KD025), 5 nM of TGF-β2-treated 3-D HCSF spheroids were prepared during a 6-day culture and subjected to a qPCR analysis for *COL1*, *COL4*, *COL6*, and *FN*. Levels of the mRNA expression were plotted. Experiments were carried out in duplicate using fresh preparations (*n* = 15, each). * *p* < 0.05, ** *p* < 0.01, *** *p* < 0.005, **** *p* < 0.001.

**Figure 8:**
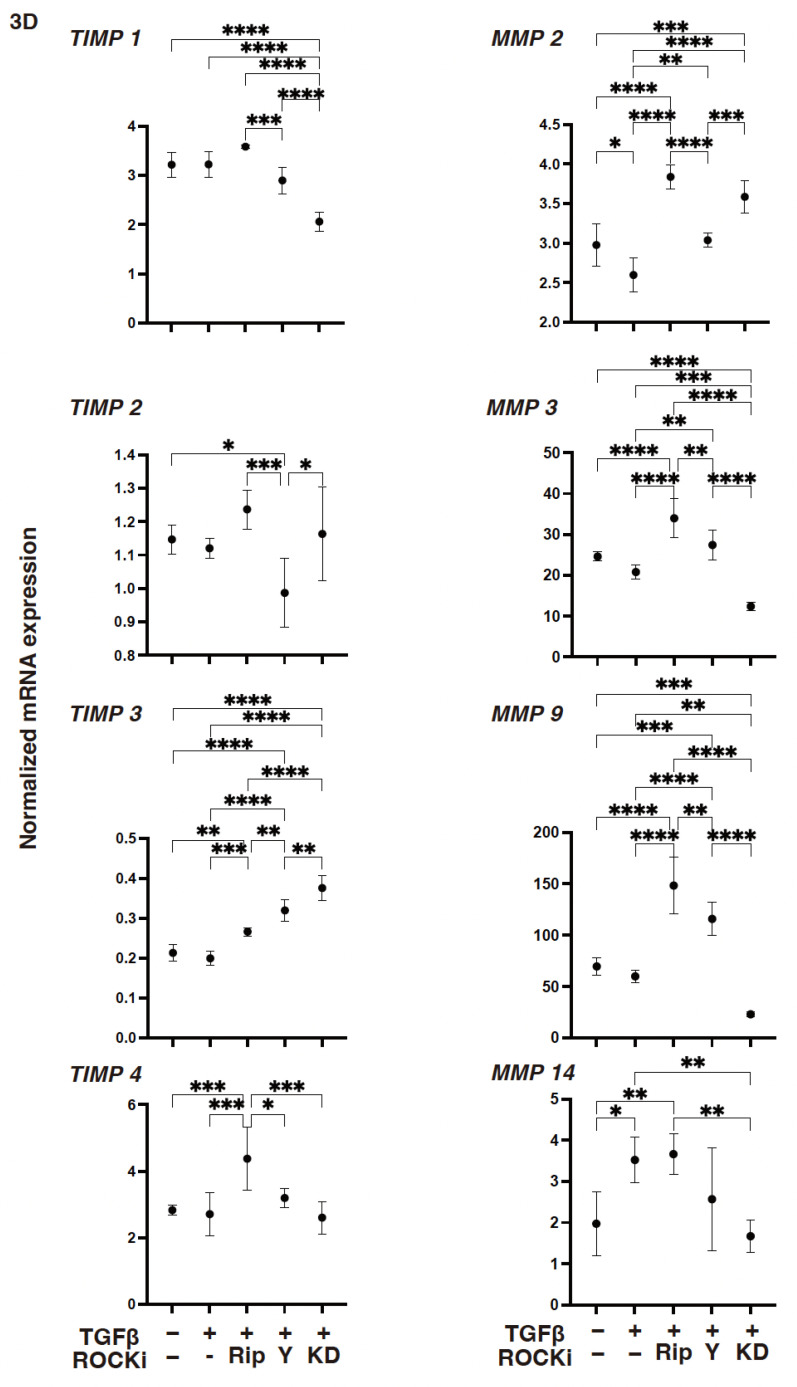
The mRNA expression of *TIMPs (1–4)* and *MMPs (2*, *3*, *9*, *14)* in TGF-β2-treated 3-D-cultured human corneal stroma fibroblasts (HCSFs) in the absence or presence of ROCK-is. In both the administered or not administered with 10 μM of ROCK-i (Rip, Y27632 or KD025), 5 nM of TGF-β2-treated 3-D HCSF spheroids were prepared during a 6-day culture and subjected to a qPCR analysis for *TIMPs (1–4)* and *MMPs (2*, *3*, *9*, *14)*. Levels of the mRNA expression were plotted. Experiments were carried out in duplicate using fresh preparations (*n* = 15, each). * *p* < 0.05, ** *p* < 0.01, *** *p* < 0.005, **** *p* < 0.001.

**Figure 9:**
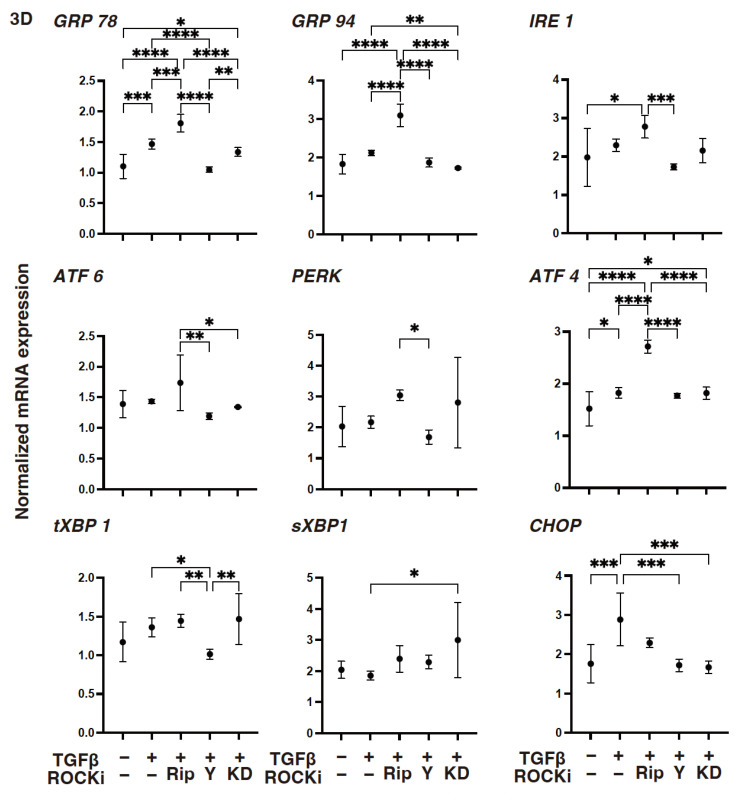
The mRNA expression of several ER stress-related genes in TGF-β2-treated 3-D-cultured human corneal stroma fibroblasts (HCSFs) in the absence or presence of ROCK-is. In both the administered or not administered with 10 μM of ROCK-i (Rip, Y27632 or KD025), 5 nM of TGF-β2-treated 3-D HCSF spheroids were prepared during a 6-day culture and subjected to a qPCR analysis for several ER stress-related genes. Levels of the mRNA expression were plotted: PERK: protein kinase RNA-like endoplasmic reticulum kinase; ATF6: activating transcription factor 6; IRE1: the inositol-requiring enzyme 1; GRP78: the glucose regulator proteins 78; GRP94: the glucose regulator proteins 94; XBP1: the X-box binding protein-1; sXBP1: a spliced XBP1; and CHOP: the CCAAT/enhancer-binding homologous protein. Experiments were carried out in duplicate using fresh preparations (*n* = 15, each condition). * *p* < 0.05, ** *p* < 0.01, *** *p* < 0.005, **** *p* < 0.001.

However, compared with the 2-D-cultured HCSF cells, TGF-β2 induced significant changes in the mRNA expressions of ECM proteins (Figure 7) and their modulators (Figure 8), and the ER stress-related factors (Figure 9) described above in the 3-D HCSF spheroids were lessened. There was a substantial decrease in the expression levels of *MMP2* and *MMP14* and an increase in the expression levels of *GRP78*, *ATF*, and *CHOP*. In addition, ROCK-is induced an alteration of the gene expressions of the TGF-β2-treated 3-D HCSF spheroids that was similar but somewhat different and exaggerated compared with those of 2-D HCSF.

As described above, there were three noteworthy developments: (1) Rip caused a significant decrease in the levels of expression of *COL1* and an increase in the levels of expressions of *COL6*, *TIMP3 TIMP4*, *MMP2*, *MMP3*, *MMP9*, *GRP78*, *GRP94*, and *ATF4*; (2) Y27632 caused a significant decrease in the levels of expression of *GRP78* and *CHOP* and an increase in the levels of expression of *COL6*, *TIMP3*, *MMP2*, *MMP3*, and *MMP9*; and (3) KD025 caused a significant decrease in the levels of expression of *COL1*, *FN*, *TIMP1*, *MMP3*, *MMP14*, *GRP94*, and *CHOP* and an increase in the levels of expression of *COL4*, *TIMP3 MMP2*, and *sXBP1*. When administered together, both ROCK1 and ROCK2 inhibitors could greatly suppress TGF-β2 induced corneal stromal EMT spreading within both their planar and spatial directions by modulating the various gene expression levels of the ECM, their modulators, ER stress-related factors, and cellular metabolic functions.

## 4. Discussion

In vitro 3-D models are known to serve as preferable in vitro models to study the pathophysiological aspects of the cornea such as wound healing, regeneration, and related diseases. These models produce a better replication of the spatial tissue microenvironment by comparison with the conventional 2-D cell culture [33,34,35]. The physiological supply of various nutrients, metabolites, oxygen, and signaling molecules could be accomplished in an indirect diffusional manner in the core of the 3-D HCSF spheroid, which is similar to the corneal stroma [36]. Therefore, such in vitro 3-D corneal models have utility in investigating various pathological conditions that include infections, injuries, fibrosis, and regenerative mechanisms in addition to allowing drug screening to evaluate their pharmacological and possible toxic effects [37,38]. In our preceding studies, we successfully developed a 3-D HCSF spheroid, and using this in vitro model, we found that pan-ROCK-i, ripasudil, as well as ROCK2-I, KD025, affected the spatial construction of 3-D HCSF spheroids in different manners [39]. In addition, we also found that the EP2 agonist omidenepag (OMD) alters the physical hardness of 3-D HCSF spheroids in response to changes in osmotic pressures [28]. Given these results, we suggest that, in addition to the 2-D cell culture, 3-D spheroid cultures could serve as a useful in vitro model to evaluate the drug-induced effects on the human corneal stroma.

ROCK1 and 2 are known to function as the important regulators of the cytoskeleton as well as the cell movement by modulating actin stress fibers and cell adhesions [40,41]. Both ROCK1 and 2 have been shown to play a pivotal role in various corneal cell functions such as cell differentiation [42], cell proliferation [43], cell adhesion [44], the reorganization of cytoskeleton [45], and cell–matrix interactions [46]. In turn, ROCK inhibitions could greatly modulate corneal wound healing by regulating cell–cell adhesion as well as contributing to the formation and maintenance of barrier integrity [43,47]. As of this writing, the roles of ROCK1 and 2 in HCSFs have been insufficiently elucidated. Recently, to elucidate this issue, the effects of pan-ROCK-i, Rip, and ROCK2-i, KD025 toward the mRNA expressions of several ECM proteins, their modulator, TIMPs, MMPs, and several ER stress-related factors of 2-D- and 3-D-cultured HCSFs as well as the physical properties of 3-D HCSF spheroids were studied [39]. It was shown that both forms of ROCK-i caused diverse effects toward 2-D and 3-D HCSFs, which suggest that ROCK1 and 2 may be differently involved in the spatial architecture of the human corneal stroma [39]. In the current study, such diverse effects of ROCK-is toward TGF-β2 induced the EMT of both 2-D- and 3-D-cultured HCSF cells were also observed.

These EMT and MET processes within the corneal stroma, however, have not yet been fully elucidated, particularly those occurring within their spatial direction. Therefore, to study this issue, we previously studied the TGF-β2-induced effects toward mRNA expressions in various samples of ECMs, their modulators, and ER stress-related molecules of 2-D- and 3-D-cultured HCSFs [9]. Interestingly, we found different TGF-β2-induced effects on 2-D- and 3-D-cultured HCSF cells. There was a significant TGF-β2-induced upregulation of most of the ECM proteins tested in the 2-D-cultured HCSF cells, but not in 3-D HCSF spheroids [9]. Identical results were again observed in the current investigation. The relatively increased levels of gene expression of *αSMA*, a marker for the myofibroblast phenotype [10] of non-treated 3-D HCSF spheroids, suggested that the EMT may already have been spontaneously evoked in the 3-D spheroid cultures. This was verified by the spontaneous adipogenic differentiation of 3T3-L1 preadipocyte spheroids [48] and by the expression of gap junction-related molecules in the H9c2 cardiomyocyte spheroids [49]. Based on these results, we speculated that such spontaneous EMTs in 3-D HCSF spheroids could represent the masking of the TGF-β2-induced alterations of various mRNA expressions in the ECM, which would include their modulators and ER stress-related factors despite significant changes in their physical properties and substantial changes in those gene expressions by all forms of ROCK-is.

We acknowledge that the present study has several limitations. Firstly, although pan-inhibitors should theoretically be more effective than ROCK2-I, KD025, some paradoxical effects of inhibition of both ROCK1 and ROCK2 were observed compared to that of ROCK2 in several experiments, including physical property measurements, cellular metabolic analysis, and gene expressions of various molecules. Interestingly, some unknown effects between pan-ROCK inhibitors and ROCK2 inhibitor KD025 were also recognized toward adipogenesis. For instance, ROCKs are known to inhibit adipogenesis, and, in turn, their inhibitors, Y-27632 and fasudil, are identified to stimulate adipocyte differentiation [50]. However, in contrast, KD025 inhibited adipogenesis, and this effect is known as ‘paradoxical anti-adipogenesis effects’ [51]. In fact, a paradoxical phenomenon between pan-ROCK inhibitors and ROCK2 inhibitor KD025 was also observed in our previous study related to adipogenesis [52]. Although, as of this writing, the exact mechanism inducing such paradoxical effects by ROCK-is remains to be elucidated. It is speculated that ROCK1 and ROCK2 signaling that regulate various gene expressions, including ECM proteins and their modulators, may be more complex than we expected. Secondly, since human specimens have biological variations, small sample sizes in the present study may lead to ambiguity in the experimental analysis. However, despite this disadvantageous situation, significant diverse pharmacological effects between ROCK inhibitors were observed in several different analyses, suggesting that our results were still important to understand the roles of ROCK1 and ROCK2 in the homeostasis of corneal stroma. Therefore, additional studies using more numbers of specimens will be required. In conclusion, the results presented in this study indicate that both forms of the TGF-β2-induced EMT occur in both the planar and spatial distributions in the corneal stroma and that these could be greatly modulated by the administration of both forms of ROCK-is. Therefore, the modulation of ROCKs could have a significant therapeutic potential toward related diseases, and our results will provide useful suggestions to understand ROCK-is-induced effects and to develop new applications and drug discoveries for corneal diseases. In support of this idea, several possible applications of ROCK-is have been expected for the therapy of corneal injury and diseases using patients with Fuchs’ endothelial corneal dystrophy (FECD) [53] and a Descemet’s stripping only model [54].

## Data Availability

The datasets used and/or analyzed during the current study are available from the corresponding author upon reasonable request.

## Supplementary Materials

The following supporting information can be downloaded at https://www.mdpi.com/article/10.3390/biomedicines12122784/s1, Table S1: Sequences of qPCR primers.
