# Supplementary material for: RHO-Associated Coiled-Coil-Containing Protein Kinase Inhibitors Significantly Modulate the Epithelial–Mesenchymal Transition Induced by TGF-β2 in the 2-D and 3-D Cultures of Human Corneal Stroma Fibroblasts
Source: Biomedicines. 2024 Dec 6;12(12):2784. doi: 10.3390/biomedicines12122784 (PMC11673340; doi:10.3390/biomedicines12122784)
Supplement: Supplementary file 1 [file biomedicines-12-02784-s001.zip › biomedicines-3346909-supplementary.pdf]

Supplementary Table S1

Sequences of qPCR primers are shown.

**Gene**	**Forward primer (5' to 3')**	**Reverse primer (5' to 3')**
36B4	TGTCTGCTCCCACAATGAAAC	TCGTCTTT AAACCCTGCGTG
COL1A1	TTCTGTACGCAGGTGATTGG	GACATGTTCAGCTTTGTGGAC
COL4A1	TGAGTCAGGCTTCATTATGTTCT	AGAGAGGAGCGAGATGTTCA
COL6A1	GTGAGGCCTTGGATGATCTC	CCTCGTGGACAAAGTCAAGT
FN	TTTGACCCCTACACAGTTTCC	TGACCACTTCCAAAGCCTAAG
TIMP1	GCTTGGAACCCTTTATACATCTTG	CCTTCTGCAATTCCGACCT
TIMP2	TGTGGTTCAGGCTCTTCTTC	GACGTTGGAGGAAAGAAGGA
TIMP3	CGGTACATCTTCATCTGCTTGA	CCTTCTGCAACTCCGACATC
TIMP4	GTTGCACAGATGGATGAAGAC	GGTTTGAGAAAGTCAAGGATGTTC
MMP2	GTGCAGCTGTCATAGGATGT	TCCACCACCTACAACTTTGAG
MMP3	TGAGTGAGTGATAGAGTGGGT	TGAACAATGGACAAAGGATACAAC
MMP9	CGTCGAAATGGGCGTCT	ACATCGTCATCCAGTTTGGTG
MMP14	CTTGAATTCCTAGACCGCTGT	TTCGCCGACTAAGCAGAAG
Atf4	ATGACCGAAATGAGCTTCCTG	CTGGAGAACCCATGAGGTTTG
Atf6	TCAGACAGTACCAACGCTTATGC	GTTGTACCACAGTAGGCTGAGA
Chop	GGAGAACCAGGAAACGGAAAC	TCTCCTTCATGCGCTGCTTT
Grp78	CATCACGCCGTCCTATGTCG	CGTCAAAGACCGTGTTCTCG
Grp94	CTGGGACTGGGAACTTATGAATG	TCCATATTCGTCAAACAGACCAC
Ire1	TTTGGAAGTACCAGCACAGTG	TGCCATCATTAGGATCTGGGA
Perk	ACGATGAGACAGAGTTGCGAC	AATCCCACTGCTTTTTACCATGA
tXbp-1	AGTAGCAGCTCAGACTGCCA	CCTGGTTCTCAACTACAAGGC
sXbp-1	GGTCTGCTGAGTCCGCAGCAGG	GGGCTTGGTATATATGTGG
